# Auricular or body acupuncture: which one is more effective in reducing abdominal fat mass in Iranian men with obesity: a randomized clinical trial

**DOI:** 10.1186/s40200-014-0092-3

**Published:** 2014-12-05

**Authors:** Mahsa Darbandi, Sara Darbandi, Ali Akbar Owji, Pooneh Mokarram, Majid Ghayor Mobarhan, Majid Fardaei, Baxiao Zhao, Hamid Abdi, Mohsen Nematy, Mohammad Safarian, Mohammad Reza Parizadeh, Mohammad Hossein Dabbaghmanesh, Parisa Abbasi, Gordon Ferns

**Affiliations:** Department of Biochemistry, Shiraz University of Medical Sciences, Shiraz, Iran; Gastroenterohepatology Research Center, Department of Internal Medicine, Nemazee Hospital, Shiraz Medical School-Shiraz University of Medical Sciences, Shiraz, Iran; School of Acupuncture-moxibustion and Tuina, Beijing University of Chinese Medicine, Beijing, China; Cardiovascular Research Center, Avicenna (Bu-Ali) Research Institute, Mashhad University of Medical Sciences, Mashhad, Iran; Department of Genetics, Shiraz University of Medical Sciences, Shiraz, Iran; Endocrine Research Center, Department of Internal Medicine, Nemazee Hospital, Shiraz Medical School-Shiraz University of Medical Sciences, Shiraz, Iran; Biochemistry of Nutrition Research Center, Faculty of Medicine, Mashhad University of Medical Sciences, Mashhad, Iran; Institute for Science & Technology in Medicine, Keele University, Hartshill, UK

**Keywords:** Acupuncture, Abdominal fat mass, Obesity, Waist circumference, Hip circumference

## Abstract

**Background:**

The prevalence of abdominal obesity is on the rise worldwide. Previous studies have indicated the higher diagnostic value of body fat distribution pattern compared with general body in abdominal obesity assessments. Several non-pharmacological methods have been suggested for obesity management, of which acupuncture has gained a great deal of research interest with promising results.

This study aimed to comparatively evaluate the effects of conventional auricular and body electroacupuncture on abdominal fat mass in obese men.

**Methods:**

The volunteers were randomly divided into four groups, including 2 interventions and 2 controls**.** This study was conducted on 80 obese volunteer men with Body Mass Index (BMI) range of 30–40 kg/m2.The intervention groups including real body electroacupuncture (A), real auricular acupuncture (C) and the control groups containing sham body electroacupuncture (B), and sham auricular acupuncture (D). All groups were in combination with a low-calorie diet for 6 weeks. BMI, Trunk Fat Mass (TFM), Waist Circumference (WC), and Hip Circumference (HC) were measured pre- and post-intervention.

**Results:**

In group A, respectively a significant reduction was shown in BMI (P < 0.005), TFM (P < 0.005), WC (P < 0.05, P < 0.005) and HC (P < 0.005) when compared with controls (Groups B and D). Interestingly, group C had significant decreases in BMI (P < 0.005), TFM (P < 0.01, P < 0.005), WC (P < 0.005) and HC (P < 0.001) after comparison with the sham. Likewise, WC (P < 0.05) and HC (P < 0.05) were significantly reduced post- intervention when compared with two treatment groups.

**Conclusions:**

In our study, acupuncture treatment (body or auricular) seemed to have an effect on reducing BMI, TFM, WC and HC. Comparison of the two types of treatment (body and auricular acupuncture) suggests that the two types of acupuncture had similar effects on reducing BMI and TFM, but body electroacupuncture is more effective in reducing WC and auricular in HC. It seems that both auricular and body electro-acupuncture combined with a low-calorie diet are efficient, simple and painless methods to reduce respectively the HC and WC fat in obese men, compared with conventional techniques.

**Trial registration:**

IRCT201201127117N2

## Background

Abdominal obesity is one of the most prevalent types of obesity with various health adverse effects [1,2]. Previous studies have demonstrated that abdominal fat distribution pattern has higher diagnostic value, compared with general body fat in the management, evaluation and treatment of obesity [3]. Abdominal obesity, central obesity, is excessive abdominal fat around the stomach and abdomen. It is associated with risk of various health disorders including hypertension, dyslipidemia, endocrine and cardiovascular diseases, type 2 diabetes, Alzheimer's disease, sexual and pulmonary dysfunctions and Erectile Dysfunction (ED) [4-12]. Weight reduction is a treatment option in obese patients with ED [9,13]. Obesity is defined as a body mass index equal to or greater than 30 kg/m^2^ [14]. There are various criteria for identifying abdominal obesity such as waist circumference (WC) (>102 cm in men and >88 cm in women); waist–hip ratio {WC/ hip circumference (HC)} (>0.9 for men and >0.85 for women) and Index of Central Obesity [6,15,16]

Various treatments are available for obesity including behavioral treatments, pharmacotherapy, surgery, dietary restrictions, regulation of physical activity, acupuncture and any combination of these methods [14]. Furthermore, Several non-pharmacological methods have been suggested for obesity management, of which acupuncture, physical activity and dietary restriction have gained a great deal of research interest with promising results [17].

Usually, body and auricular acupuncture are used in a combined technique for treatment process [17-19]. In traditional Chinese medicine, life force is known as ‘Qi’. Qi is circulating within energy pathways or ‘meridians’ throughout the body stimulation during illness [20]. Acupuncture is performed by stimulating particular points on the skin during illness to balance an excess/deficiency of Qi [20]. Some of these points are on the body and others are on the ear. Among several methods to stimulate acupoints on the body, needling is one of the most common methods. The needle can be kept for varying lengths of time and can be stimulated manually or electrically (‘electroacupuncture’) [20]. The needles used for the acupuncture are very thin so that no pain is sensed in the area of needling. However, increasing the frequency of needling to 12 times and higher in the same point makes the cite very painful [20].

Many studies have examined the effects of acupuncture on weight loss [21,22]. Previous studies have demonstrated the beneficial effects of combination of auricular and electro-acupuncture in obese people to decrease BMI and BW [18,23-25].

However, most studies conducted on the feasibility and efficiency of combined body and ear acupuncture suffer from methodological limitations, inadequate study design, or inappropriate complimentary treatment. To our knowledge, previous studies evaluating the effects of acupuncture have not considered abdominal fat mass in their assessments. Since many impotent men are obese and obese men will be usually relieved from ED when their weight is reduced, in the present study we evaluated the effects of body electroacupuncture and auricular acupuncture combined with a low-calorie diet on the abdominal fat mass in our male volunteers and then compared these two types of treatment with each other.

## Method

### Participants

Eighty men, aged 18–50 years old with a BMI between 30–40 kg/m^2^ (Table 1) who attended the Nutrition Clinic at Ghaem Hospital (Mashhad, Iran). were selected. They had received no relevant medical or drug history within the 3 months prior to the start of experiment. The exclusion criteria included diabetes, severe hypertension, heart disease, endocrine abnormalities (patients with any history of hormonal problems), allergy and other immunological disorders.

**Table 1:** **The demographic data of treatment and sham groups**

**Groups**	**Electro acupuncture**		**Auricular acupuncture**	
	**Treatment (n = 20)**	**Sham (n = 20)**	**Treatment (n = 20)**	**Sham (n = 20)**
**Age (years)**	38.0 ± 0.9	38.0 ± 1.3	39.0 ± 1.8	37.9 ± 1.5
**Height (cm)**	171.0 ± 2.5	168.8 ± 1.6	173.9 ± 1.8	175.6 ± 0.7
**Weight (Kg)**	99.6 ± 3.9	93.1 ± 4.2	102.0 ± 2.7	100.0 ± 2.7
**BMI (Kg/m** ^**2**^ **)**	33.4 ± 1.3	33.0 ± 1.5	33.4 ± 2.6	32.0 ± 3.9
**TFM (%)**	17.0 ± 1.5	16.6 ± 1.5	16.5 ± 2.2	15.6 ± 1.7
**WC (cm)**	108.2 ± 2.7	106.3 ± 2.1	108.3 ± 3.8	107.4 ± 2.4
**HC (cm)**	116.6 ± 2.9	114.6 ± 2.4	113.2 ± 3.2	112.2 ± 3.8

BMI: body mass index. TFM: trunk fat mass. WC: waist circumference. HC: hip circumference. Values are means ± SD except where indicated. Independent t-tests were used to compare treatment and Sham groups. The demographic parameters did not differ significantly between the treatment and control groups before treatment (p > 0.05).

The written consent form was obtained from all of the participants and kept in their medical records. All of the experimental procedures of the study were approved by the Ethics Committee of the Mashhad University of Medical Science Research Council

Anthropometric assessments were performed pre- and post-intervention. Based on random number tables, the participants were enrolled in the study after checking for the inclusion/exclusion criteria, and were arranged into pairs matched by age and BMI category. Using random numbers generated in Microsoft Excel (Redmond, Washington, USA), pairs were randomly divided into four equal groups (n = 20) including two treatment groups and two Sham groups. All 4 groups included 1) group A (n = 20) receiving body electroacupuncture accompanied by a low-calorie diet as the first treatment group, 2) group B (n = 20) receiving sham body electroacupuncture accompanied by the same low-calorie diet, 3) group C (n = 20) receiving auricular acupuncture in combination with a low-calorie diet as the second treatment group; and finally 4) group D (n = 20) that received sham auricular acupuncture accompanied by the same low-calorie diet. Groups B and D were organized respectively as a Sham of the group A and the group C (Table 1).

Macronutrients in volunteers were prepared with daily food. Basal metabolic rate (BMR) is basically the amount of calories required on a daily basis if people do not move at all and lose the minimal amount of energy. The Harris Benedict Formula takes the number produced by the BMR formula and multiplies it depending on the activity level. In our study, the energy expenditure was computed with the equation of Harris and Benedict. The participants consumed an isocaloric diet two weeks before starting the study, and then continued with the same low-calorie diet for 6 weeks. The isocaloric diet is a moderate-carbohydrate, moderate-fat diet that allows volunteers to eat whatever they want as long as they consume the same amount of carbohydrates, proteins and fats daily. The low-calorie diet contained 500-kcal energy per day. The resting energy expenditure was computed with the equation of Harris and Benedict [26] that is used to set the quantity of energy per day for each participant in case and control groups to those matched with them. The isocaloric diet and the 6-week dietary program for each participant were planned by a nutritionist based on the participant’s energy expenditure. The participants were followed up every week.

### Interventions

#### Body electroacupuncture treatment in group A

The research team identified standardized body acupuncture points according to the traditional Chinese medical practice and clinical experience. Based on a short interview, the team classified participants as having an excess (phlegm-dampness or phlegm-heat) or deficiency (Spleen/Stomach Qi Deficiency or Primary Qi Deficiency) pattern [20]. Each of these patterns was manifested with very different signs and symptoms differentiated by the practitioner based on age, diet, emotions, digestion, lifestyle, family medical history and accompanying signs and symptoms. Eight acupuncture points on the abdomen, including Tianshu ( ST-25) on both sides, Weidao (GB-28) on both sides, Zhongwan (REN-12) Shuifen (REN-9) Guanyuan (REN-4) Sanyinjiao (SP-6), and additional points are Quchi ( LI-11) and Fenlong (ST- 40) for excess mood (patients with higher energy), and Qihai (REN-6) and Yinlingqau (SP-9) for deficiency mood (patients with lower energy) on both legs were selected.

A modified common acupuncture needling manipulation and 2 normal electric output with 4 lines from the electric acupuncture machine (Ying Lee, KWD 808) to 4 needles (each output connected to the needles of ST25 and GB28 of the same side) used for 20 minutes. After sterilizing the acupoints with 75% alcohol preparation pads, the acupuncturist inserted the needles into the sites while the participant was lying in the prone position. The needles were maintained on the body for 20 minutes.

In this group, the 3.8 cm stainless steel acupuncture needles (Acupuncture needles, Beijing Zhongyan Taihe Medicine, Beijing, China) were inserted to a depth of approximately 2.5 cm after skin sterilization. The needles were connected to an electrical stimulator. The stimulating current was generated with a pulse generator (30–40 Hz, dense-disperse wave, 390 μS square pulse) and at a maximal tolerable intensity, 500 Ω (12–23 V) (a strong but not painful sensation to the patient). Each acupuncture treatment lasted for 20 minutes.

All subjects received two treatment sessions per week for a total of 6 weeks. All needling was done by an expert acupuncturist.

Standardized body acupoints were selected based on the Chinese medical practice and clinical experience. Participants were classified as having the excess or deficiency pattern [20] according to a short interview.

#### Sham body electroacupuncture in group B

For acupoints on the REN meridian, the needles were inserted as superficially as possible relative to the location 0.3 cun laterally to the real location. Other points were located 0.5 cun cranially and 0.5 cun laterally from the real location, with needling as superficial as possible. We used disconnected electric output lines, which looked the same as the real lines, from the acupuncture machine to 4 needles (each output connected to the needles of ST25 and GB28s of the same side) for 20 min [20] so the study was just blind for volunteers and other colleagues. This method was used for the group B as a Sham of group A. No major adverse effects were observed in any of the participants.

#### Auricular acupuncture treatment in group C

Standardized acupoints were selected on the external ear according to the Chinese medicine and clinical experience: Shenmen (TF4), stomach (CO4), hungry point, mouth (CO1), center of ear (HX1), and Sanjiao (CO17). Acupuncture in the treatment group was applied with routine ear pressing plasters (Vaccaria ear seeds, Beijing Zhongyan Taihe Medicine, Beijing, China). After sterilizing the acupoints with 75% alcohol preparation pads, the acupuncturist inserted the ear-pressing plaster with the seed into the acupoints on both ears in each treatment and kept on the ear for 3 days [20]. All participants were requested to apply pressure to the auricular points before eating. They received two treatments per week for a total of 6 weeks. The procedure was performed by an expert acupuncturist and no adverse effects were reported. This method was used for group C as a treatment group.

#### Sham auricular acupuncture in group D

Group D, as the Sham of group C, received sham auricular acupuncture using placebo plasters (ear plasters without seeds (Vaccaria ear seeds so the study was just blind for volunteers and other colleagues. Beijing Zhongyan Taihe Medicine, Beijing, China). The hip (AH5), spleen (CO13), nose, and esophagus (CO2) points were used in this method. The treatment procedure was the same as in group C.

### Statistical analysis

Statistical package for social sciences (SPSS) software (version 16, Chicago, IL, USA) was applied for all statistical analyses. Descriptive statistics (mean, standard deviation and interquartile range) were determined for all variables. Data were checked for normality with the Kolmogorov-Smirnov test. ANOVA was used to compare the mean differences of variables in four groups. In all statistical analyses, a P value of 0.05 was set as significant.

### Outcome measures

#### Anthropometric measurement

In all participants, anthropometric parameters including the height, waist and hip circumference were measured using standard protocols. BMI and TFM were measured with a Tanita BC-418 body composition analyzer (Tanita, Tokyo, Japan) based on a standard protocol [27]. Variables were recorded at the beginning of the study and the day after treatment. All measurements were performed between 7.00 and 9.00 AM. The participants had abstained from food since the previous evening. All variables were measured and recorded with standard procedures.

#### Blood samples

In all groups, 12 hour fasting blood samples were collected between 7.00 and 9.00 AM with the standard methods twice: the first time, at the beginning of the study and the second time, the day after treatment.

The biochemical test results such as Cr, Albumin, Uric acid, FBS, LDL, HDL,WBC, RBC of the participants were measured for 2 times to notice the exclusion criteria at the start of study and monitor side effects of the treatment during the acupuncture.

## Results

**Figure 1:**
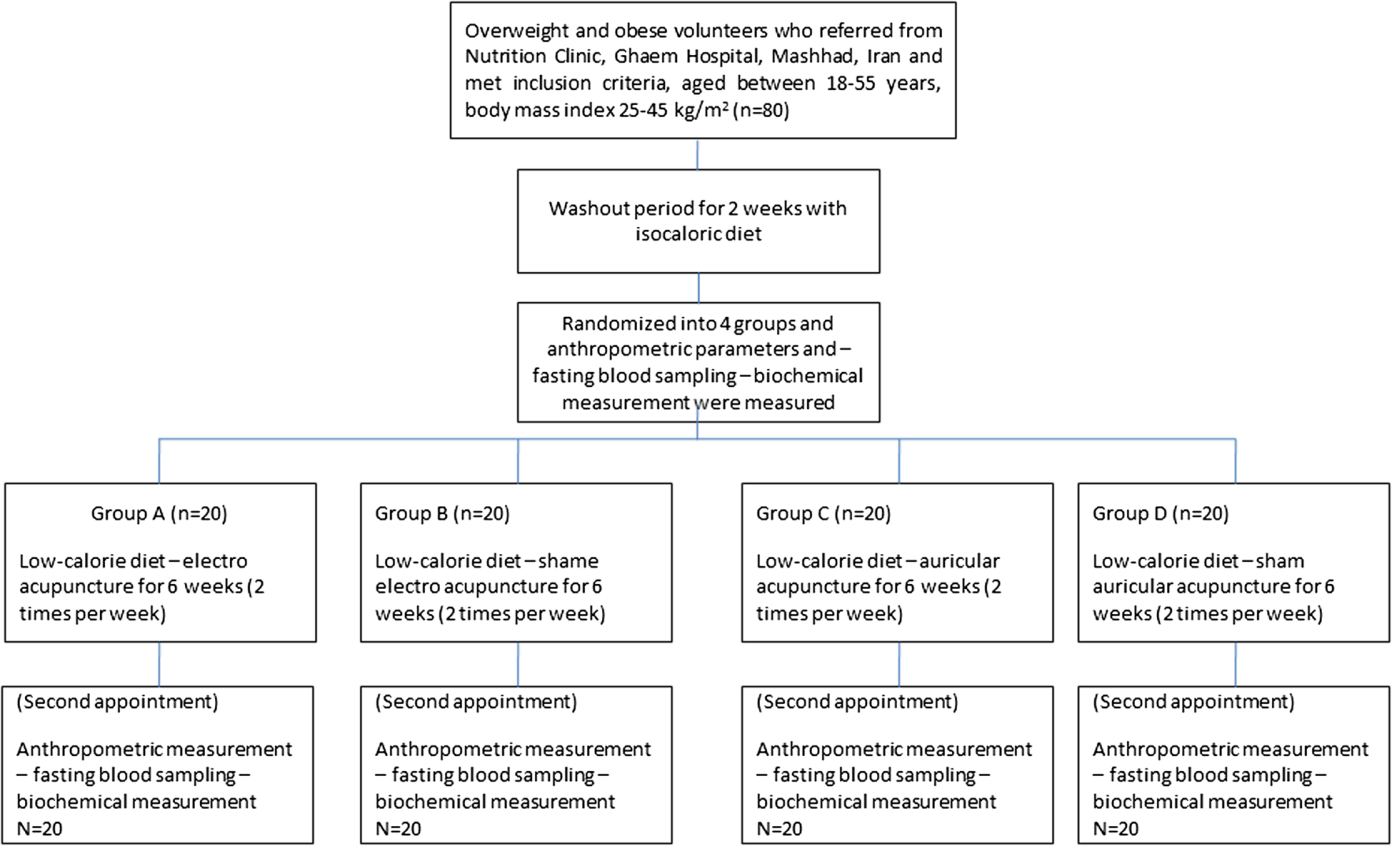
**Flowchart related to the study design of this work.**

A total of eighty participants fulfilled the inclusion criteria. The study design is illustrated in Figure 1. The analysis showed that age, height and anthropometric parameters were not significantly different between treatment and sham groups (P > 0.05) at baseline. Clinical characteristics of the participants are summarized in Table 1.

Group A, implementing body electroacupuncture with low-calorie diet, produced a significant reduction in the mean difference of BMI (P < 0.005), TFM (P < 0.005), WC (P < 0.05, P < 0.005) and HC (P < 0.005) after 6 weeks of intervention when compared with the controls (Groups B and D); the P values are shown respectively.

Interestingly, group C had significant decreases in BMI (P < 0.005), TFM (P < 0.01, P < 0.005), WC (P < 0.005) and HC (P < 0.001) after combined treatment of auricular acupuncture with a low-calorie diet for 6 weeks when compared with Group D. Furthermore, theis group showed significant reductions in BMI (P < 0.005), TFM (P < 0.01) and HC (P < 0.005) after 6 weeks of treatment but not in WC (P > 0.1) when compared with Group B. Likewise, WC (P < 0.05) and HC (P < 0.05) were significantly reduced post-intervention when compared with two treatment groups. Levels of different parameters are summarized in Table 2.

**Table 2:** **Changes observed in the four groups over the study period**

**Groups**	**Electro acupuncture**		**Auricular acupuncture**	
	**Treatment (n = 20)**	**Sham (n = 20)**	**Treatment (n = 20)**	**Sham (n = 20)**
**Changes**	**1** ^**st**^ **-2** ^**st**^	**1** ^**st**^ **-2** ^**st**^	**1** ^**st**^ **-2** ^**st**^	**1** ^**st**^ **-2** ^**st**^
**BMI (kg/m** ^**2**^ **)**	1.1 ± 0.2	0.6 ± 0.2^ab^	1.0 ± 0.4	0.7 ± 0.2^ab^
**TFM(%)**	1.4 ± 0.5	0.8 ± 0.3^ab^	1.2 ± 0.6	0.5 ± 0.2^ab^
**WC (cm)**	5.5 ± 1.1^b^	4.3 ± 1.7^a^	4.4 ± 1.0^a^	0.9 ± 1.7^abc^
**HC (cm)**	4.5 ± 1.2^b^	2.5 ± 0.8^ab^	5.3 ± 0.8^a^	0.2 ± 1.1^abc^

**1** ^**st**^ **-2** ^**st**^ **: the treatment period.** 1^st^ measurement: measurement of the below parameters at the beginning of the study. 2^st^ measurement: measurement of the below parameters one day after treatment. BMI: body mass index; TFM: Trunk fat mass: WC: waist circumference; HC: hip circumference. Values are means ± SD. ^a^mean significant changes in comparison with first treatment and ^b^means significant changes in comparison with second treatment group. ^c^means significant changes in comparison with first sham group. In all statistical analyses a P value ≤ 0.05 was set as significant.

Because the data were normally distributed, ANOVA was used to compare the mean differences of variables in four groups.

## Discussion

Our results indicated that auricular and body acupuncture respectively are more effective in reducing HC and WC.This study showed a remarkable reduction in WC and HC in the obese men in real auricular acupuncture, whereas the sham auricular acupuncture did not show any significant effects. All the participants who received real and sham body electroacupuncture showed a significant reduction in WC and HC. However, these changes were more evident in real group. On the other hand, reductions in BMI and TFM were observed in real and sham groups but real treatment groups showed a significant reduction than the sham group.

Some previous studies showed the efficiency of acupuncture in weight loss [7,12-14,18,20,28-32]. In agreement with previous studies, our findings showed the efficiency of acupuncture in abdominal fat mass [33]. It is consistent with our results indicating the greater efficacy of body acupuncture therapy for reduction of anthropometric parameters in cases when compared with controls. A randomized crossover trial in obese women reported significant changes in anthropometric parameters by electroacupuncture when compared with sit-up exercise [24]. The same results have been found by other studies in women [23,30]. No specific studies have been conducted on obese men and this is the first study on male abdominal obesity. However, other studies have reported no significant effect of acupuncture in the treatment of obesity [31,32,34].

In this study, auricular acupuncture had remarkable effectson reducing HC, but it was not shown in body acupuncture. Also it was indicated that body acupuncture is more effective in reducing WC.

It should be noted that there are some limitations in the study, including very short follow up and very small sample size. To confirm this finding, this study should be conducted on a larger number of subjects of both sexes.

Acupuncture appears to have the ability to improve the mood by increasing the release of neurotransmitters [35] and suppress the appetite by serotonin and endorphin-induced decreases in stress and depression [36,37], whereas this effect was not seen by exercise and diet. In addition, it is shown that application of electroacupuncture at Zusanli (ST-36) and Neiting (ST-44) of threat caused an increase in the electrical activity of the ventral-medical hypothalamus in the obese rat, leading to activation of the satiety center [38]. Waist circumference is related to the subcutaneous fat tissue of the abdomen. Higher effects of body acupuncture on the lipolytic activity and enhancing lipid metabolism could be attributed to the direct effects of body acupuncture on redistribution and lyses of fat tissue, thereby reducting the waist circumferences [23,39,40].

Reductions in abdominal fat mass reported in this study confirm this statement. Moreover, we observed that participants who received acupuncture showed a larger decrease in abdominal fat mass than their counterparts in the sham group.

As mentioned earlier, several studies have shown that abdominal obesity in men correlates with some important diseases [4-13,41,42]. Therefore, abdominal fat reduction can reduced these complications [12]. Our study suggests that reduction in WC and HC may be affected by a decreasein BMI and TFM. The decrease in HC and WC was significantly greater in the treatment group as compared to the sham group (Table 2). Comparison of the two types of treatment (body and auricular acupuncture) suggests that the two types of acupuncture had similar effects on BMI, TFM, but body electroacupuncture is more effective in reducing WC and auricular in HC. The fact that reductions in TFM, WC and HC were seen in both real and sham body electroacupuncture groups but not in the sham auricular acupunctures group implies that in body acupuncture treatment, needling in the areas away from the main points on the body like body electroacupuncture was beneficial for reducing weight and abdominal fat mass although this was not reduced to the size of the real group. This was probably due to physical or psychological effects of needling on the body.

Although under our working conditions it may not have a good efficacy, but the method can be used in future studies to be optimized and increase the efficacy. Considering that the waist-hip ratio was decrease in this study, this treatment may contribute body fat distribution. This notion may be used for future studies.

The discrepancies may be due in part to the difference in participant demographics and anthropometric characteristics as well as different durations of the treatment courses. Conducting further studies is necessary to profoundly evaluate other specific points for body electroacupuncture which cause the additive effects on WC and HC due to needling and correct points. In addition, further studies in male and female infertility are recommended. However, further research is needed to elucidate the mechanism by which body electroacupuncture and auricular acupuncture affect abdominal fat mass.

## Conclusion

According to our findings, it seems that both body electroacupuncture and auricular acupuncture in combination with dietary treatment are beneficial complementary treatments in losing weight and reducing abdominal fat mass in obese men. In addition, we found that needling in the areas away from the main points on the body had effects on anthropometric characteristics that can be attributed to the physical or psychological effects of needling on the individuals. Although these effects can be achieved by other physical or chemical modalities, lack of adverse events makes acupuncture an appropriate or adjunctive treatment option for obesity control. Therefore, although many complementary therapies have been proposed for treatment of obesity, few are truly of therapeutic importance for abdominal fat mass reduction. To the best of our knowledge, this is the first study that evaluates the effects of two types of acupuncture on abdominal fat mass and compares the effectiveness of these treatments on obese men.

Since auricular acupuncture with a low-calorie diet is an effective, painless and simple method, we suggest these methods as a complementary treatment respectively to reduce HC and WC fat mass in men. But to confirm this finding, this study should be performed on a large number of subjects.

## Abbreviations

BMI: Body mass index
TFM: Trunk fat mass
WC: Waist circumference
HC: Hip circumference
BMR: Basal metabolic rate
